# A systematic review of coping and pre-death grief among dementia family caregivers

**DOI:** 10.1017/S1478951525000082

**Published:** 2025-04-02

**Authors:** Yi-Qi Wangliu, Run-Ping Che

**Affiliations:** 1 Department of Social Work, The Chinese University of Hong Kong, Shatin, NT, Hong Kong SAR, China; 2 School of Ethnology and Sociology, Yunnan University, Kunming, mainland, China

**Keywords:** Dementia, caregivers, grieving, palliative care, coping

## Abstract

**Objectives:**

Research on grief among family caregivers of individuals with dementia has seen a notable increase. Our objective was to synthesize the relationship between coping factors and pre-death grief (PDG).

**Design:**

(Prospero protocol: CRD42024560208) We conducted a systematic review of literature from PubMed, Web of Science, Scopus, PsycInfo, and Medline up to July 2024. Included studies encompassed quantitative, qualitative, and mixed methods approaches. During the study selection process, we excluded data on intervention effectiveness and studies not published in English. The quality of the studies was evaluated using the Mixed Methods Appraisal Tool. Evidence was summarized narratively.

**Participants:**

Participants in this study are family caregivers who take care of dementia patients.

**Methods:**

We included data from 12 studies in our analysis. The majority of these investigations were carried out in Western countries. The research primarily involved spousal or adult child caregivers and centered on PDG. We included validated measures of PDG in each study.

**Significance of results:**

Among the reviewed studies, five reported on coping strategies, while seven addressed coping resources. Overall, the findings indicated that the application of coping strategies, specifically positive coping strategies, is effective in alleviating PDG and mitigating the effects of caregiving burden on PDG. Coping resources – including self-efficacy, sense of coherence, and support from friends and family – appear to have a beneficial impact in reducing PDG. Additionally, the quality of relationships with friends and family members was found to be a significant factor. Moreover, spiritual and religious beliefs, along with community faith, have been identified as crucial elements in alleviating grief experienced by caregivers.

**Conclusion:**

Knowing what coping strategies and resources are beneficial to decrease PDG experiences among dementia caregivers.

Grief is commonly conceptualized as the psychological response to a disruption in a significant attachment due to death or loss (Neimeyer et al. 2002; Stroebe et al. 2010). Caregivers exhibit a high prevalence of stress, grief, and depression prior to the physical death of individuals with dementia (Adams & Sanders, 2004; Joling et al. 2012; Ott et al. 2010; Pinquart & Sorensen, 2004); however, they often fail to recognize the symptoms associated with pre-death grief (PDG) (Marwit & Meuser, 2001; Silverberg 2007). The experience of PDG intensifies as dementia progresses (Adams & Sanders, 2004; Chan et al. 2013; Meuser & Marwit, 2001; Ott et al. 2010), alongside a decline in caregiver health (Holley and Mast 2010) and an increased sense of burden (Chan et al. 2013; Holley and Mast 2009; Wangliu and Chen 2024).

Noyes et al. (2010) summarized caregiving and relationship losses as significant components of PDG. Caregiving losses refer to the sacrifices and dedication of caregivers, including losses of social contact, job opportunities, personal freedom, identity, and well-being. Relationship losses pertain to the emotions and relationships between the caregiver and the patient, such as the loss of intimacy, communication, mutual support, or changes in the relationship dynamics. These losses can occur throughout the entire dementia caregiving process, affecting the mutual relationships between caregivers and patients (Large and Slinger 2015).

The proposed grief-stress model of care-giving proposed by Noyes and colleagues integrates PDG into the framework of stress and coping model (Noyes et al. 2010) This model argues that coping is related to PDG and could be mediators between stressors and PDG. However, empirical studies are scarce to prove this argument. Considering coping is the most essential part during the grieving, how coping is related to grief should be paid great attention.

Most research provides theoretical frameworks for understanding caregiver outcomes through the lens of the Transactional Model of Stress and Coping (TSC; Lazarus and Folkman 1984). In this model, coping is characterized as a dynamic process involving “constantly changing cognitive and behavioral efforts to manage specific external and/or internal demands… appraised as taxing or exceeding [personal] resources” (p. 141). Central to this process are coping strategies and coping resources. Coping strategies encompass the behavioral and cognitive tactics employed to address crises, stress, and various internal or external demands (Pinquart & Sörensen, 2004). These strategies are generally categorized into problem-focused approaches, which involve actions that alter the relationship between the individual and their environment; emotion-focused approaches, which modify the meaning of that relationship through methods such as avoidance, distraction, and minimization; and cognitive approaches, which affect stress and emotion by re-evaluating the person-environment relationship (Lazarus and Folkman 1984: 77). Additionally, Haley et al. (1987) found that both appraisal and coping responses serve as significant predictors of outcomes for caregivers of adults with dementia.

Numerous studies have employed the Brief Cope scale to evaluate coping strategies among dementia caregivers. This scale is commonly employed in healthcare environments to evaluate patients’ emotional responses to critical situations (Baranauskas et al. 2024). It assesses individuals’ coping mechanisms across a wide range of adversities, including cancer diagnoses, heart failure, injuries, assaults, natural disasters, financial stress, and mental health challenges. The scale effectively identifies an individual’s predominant coping styles, as reflected in scores from three subscales: Problem-Focused Coping, Emotion-Focused Coping, and Avoidant Coping. Additionally, it measures various dimensions of coping, including Self-Distraction, Denial, Substance Use, Behavioral Disengagement, Emotional Support, Venting, Humor, Acceptance, Self-Blame, Religion, Active Coping, Use of Instrumental Support, Positive Reframing, and Planning.

In addition to coping strategies, the concept of coping encompasses coping resources (Lazarus and Folkman 1984). Prior research has concluded that coping resources at the individual, family, and social levels are essential for managing the stressors associated with caregiving (Morrow-Howell and Wang 2013). The accessibility of these resources can significantly influence both the quantity and quality of coping mechanisms available to caregivers. It has been posited that personal coping resources include enhanced self-perception and positive transformations in life outlook (Li et al. 2022). Moreover, caregivers may receive instrumental and emotional support from family or friends, which can assist in mitigating the challenges linked with caregiving (Creasey et al. 1990). Research specifically highlights that families play a vital role in providing support to older adults with chronic cognitive and physical impairments living in non-institutional settings. Family members provide tangible assistance, emotional support, and informational aid (La Fleur and Salthouse 2017). Previous studies have identified a correlation between favorable caregiving outcomes and the presence of social support (Nemcikova et al. 2023).

Beyond familial systems, the wider community significantly contributes to the understanding of care for individuals diagnosed with mental illness (Marsh 2000; Stein and Wemmerus 2001). Common support services tailored for dementia caregivers include providing information and resources related to the condition of their elderly relatives or friends. Emotional support can be accessed through support groups or individual counseling. Respite services are often available, whether at home, in daycare centers, or through temporary institutional admissions. Furthermore, home care services can assist frail seniors with activities of daily living (ADLs) and instrumental ADLs, thereby reducing some of the caregiving burden (Savard et al. 2006).

Aranda and Knight (1997) suggested that ethnicity plays a role in the cultivation of distinct cultural values that directly impact caregiving practices. This incorporates traditional cultural values as structural status variables (where ethnicity primarily signifies membership in a disadvantaged minority group, often intertwined with lower socioeconomic status), and it offers a fresh interpretation of the impact of cultural values on stress and coping mechanisms. Specifically, filial piety is a fundamental principle in Confucianism that originates from Chinese culture (Yeh 2006). It encompasses a set of moral norms, values, and practices centered around demonstrating respect and care for one’s parents. This cultural resource profoundly influences individuals’ behavior and forms the basis for their commitment to providing caregiving for elderly parents, ultimately impacting their overall well-being (Yeh 2006).

Research focused on coping strategies specifically in the context of dementia caregiving began in the mid-1980s (Haley et al. 1987), gained considerable momentum in the early 1990s, and continues to evolve (Papastavrou et al. 2011). A consensus has developed across numerous studies, emphasizing the vulnerability of caregivers to psychiatric morbidity and the variability in their coping resources. Nevertheless, more recent research has yielded findings that diverge from earlier conclusions (Baker et al. 2010). Most studies focus on the relationship between coping strategies and various aspects of health and well-being, such as depression, anxiety, life satisfaction, perceived physical health, burnout, general distress, and subjective burden (McGonaghy & Caltabiano, 2005). For instance, a correlation has been identified between depression, coping mechanisms, and the perceived burden experienced by caregivers (McConaghy and Caltabiano 2005).

Some researchers have offered a more nuanced examination of various coping responses and their correlation with depression, arguing that not all coping strategies play a positive role in caregiving outcomes (Bannon et al. 2022). Recent investigations have begun to explore the relationship between coping and PDG within this context. For example, it has been demonstrated that active coping significantly mediates the relationship between caregiver burden and PDG among caregivers of individuals with dementia (Wangliu and Chen 2024). In contrast, caregivers who employ less active coping strategies may experience increased levels of PDG.

Despite numerous studies examining the connections between coping, depression, and anxiety, there remains a paucity of empirical research focused on the dynamic and evolving nature of coping efforts in relation to PDG in the context of dementia caregiving. Therefore, this research aims to investigate how coping – encompassing both coping resources and strategies – is related to PDG.

## Methods

The review protocol CRD42024560208 was registered with the PROSPERO international prospective register of systematic reviews and adhered to PRISMA guidelines). We included qualitative, quantitative, and mixed-methods studies during the full-text review phase in a recent decade.

### Inclusion criteria

• Type of studies: We include both quantitative, qualitative and mix-methods studies.

• Research focus: We focus on associations between PDG and coping, encompassing coping strategies and resources among dementia family caregivers.

• Participants: Family non-paid caregivers (aged 18 years or older) of individuals with dementia were included.

• Setting: Participants provided care or support for individuals living with any type and severity of dementia in community settings or long-term care facilities. Bereaved caregivers were also included.

### Exclusion criteria

• Excluding intervention studies

• Excluding studies not written in English

• Excluding papers in which participants are paid/professional carers

• Excluding gray literature (research produced outside of traditional publishing and distribution channels and excluded at full-text review).

## Search strategy

### Data search and selection criteria

To identify relevant articles published in reputable scholarly journals and written in English, a systematic search was conducted across multiple electronic databases, including PubMed, Web of Science, Scopus, PsycINFO, and Medline. This search spanned from April 2024 to July 2024. The following key terms were used in the abstracts: terms related to dementia (dement* or alzheimer* or ADRD or vascular or (lewy* adj2 bod*) or FTLD or frontotemp* or “organic brain disease” or “organic brain syndrome” or “major cognitive disorder”), family caregivers (caregiv* or carer* or famil* or relative* or kin or spouse* or adult child*), pre-death grief (pre-death grief* or pre-death loss* or pre-death bereav* or *pre-death mourning* or *anticipatory grief*), and coping (coping or coping strategies or coping resources or coping behavior) (see Supplementary Material Section 1). Additionally, manual searches of reference lists from relevant reviews were performed to uncover potentially eligible studies that may have been overlooked in the electronic searches. Conversely, studies classified as abstract-only articles, case reports, conference papers, commentary articles, gray literature, protocols, review articles, systematic reviews, and meta-analyses were excluded.

### Screening

Two reviewers evaluated the relevance of article titles and abstracts in relation to the research question, adhering to the established inclusion and exclusion criteria. Articles whose abstracts fulfilled these criteria underwent full-text assessment, followed by a manual review of the bibliographies of the included studies. Ultimately, those meeting the criteria were selected for inclusion. A third reviewer was designated to resolve any conflicts arising from disagreements between the initial reviewers.

### Data extraction

**Figure 1.**
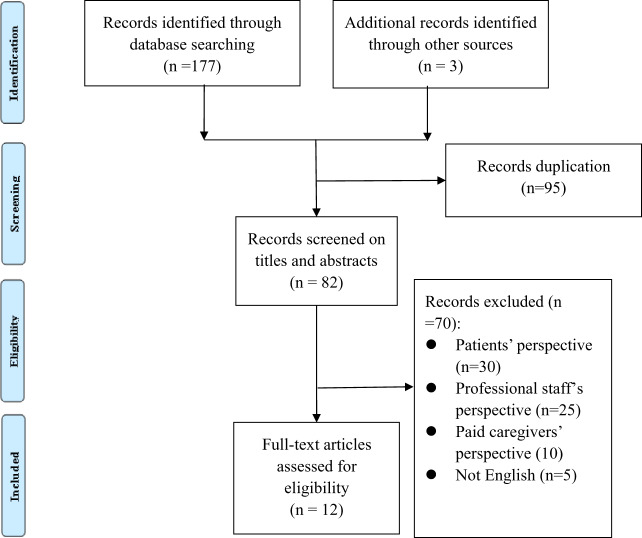
Flow diagram of the selection process.

In cases where discrepancies arose, a third colleague was consulted to aid in reaching a consensus between the first two reviewers. The complete texts of 82 articles were scrutinized for eligibility. Of these, 73 articles were subsequently excluded. The reasons for exclusion included studies from the perspectives of patients, professionals and paid caregivers, not specific to informal caregivers (Figure 1). Finally, from the 12 remaining studies, we extracted data on the characteristics of the studies, including the author(s), and year, research design, research location, study sample, and main findings on coping influencing PDG.

The characteristics of the studies were extracted by two authors and compiled into a table specifically designed for this review. The extracted data encompassed the country of origin, study design, details regarding the PDG measurement tools employed, participant characteristics, and outcomes, including the coping strategies and resources utilized by caregivers to manage PDG. Additionally, two authors independently verified 20% of the data extraction for accuracy.

### Data analysis

The Cochrane framework was employed to summarize study characteristics and synthesize data. During the protocol phase, specific research questions were outlined, and proposed analyses were established. Evidence for Question 1 that how coping strategies is related to PDG was synthesized by summarizing the associations between varying types of coping strategies and PDG, while Question 2 that how coping resources is related to PDG was addressed by summarizing the associations between varying levels of coping resources and PDG. First, we undertook a comprehensive assessment of the included studies to explore similarities and divergences in their findings. Subsequently, we conducted the narrative analysis, resulting in the collection of findings of eligible studies and evaluate the strengths of the findings of the studies (Dehkordi et al. 2021). Then, we summarized the results and grouped the findings according to the same research question. Due to high heterogeneity (study populations, data sources, designs, and statistical methods) and a small number of studies, we did not conduct a meta-analysis of the data. The PRISMA checklist was employed.

After eliminating duplicates, we identified distinct citations that met our inclusion criteria for full-text review. We included quantitative, qualitative, and mixed-methods studies in our analysis.

### Sample size and characteristics

The majority of studies were conducted in Western countries, such as USA, UK, and Australia, with only one study taking place in China. Nine quantitative studies, two mix-method studies and one qualitative study. These investigations primarily involved caregivers, specifically spouses or adult children, and focused on the phenomenon of PDG. Grief assessments include The Marwit and Meuser Caregiver Grief Inventory, Caregiver Grief Questionnaire, Two-Track Dementia Grief Questionnaire, Caregiver Grief Scale and Inventory of Complicated Grief. Among these studies, five reported on coping strategies, while seven detailed coping resources. Each article included in the final sample underwent a rigorous reading and annotation process. Data were subsequently extracted from these articles to construct a comprehensive table that delineates various study characteristics (see Table 1). The extracted data encompassed information regarding the aims and design of each study, participant characteristics such as sample size, mean age, and gender, the country of research, and key findings. The results of each study were analyzed and synthesized using narrative synthesis, a method articulated by Popay et al. (2006). This technique enabled the systematic organization of findings from the included studies, facilitating the presentation of pertinent information, relationships, and conclusions.

**Table 1.** Key findings

Study	Country	Study design	Sample size and characteristics	Sampling	Inclusion	Grief assessments	Coping assessments	Key findings
Chan et al. 2017	China	Quantitative design	N = 120; 66.7% female; mean age: 55.46 (14.89)	Purposeful sampling, children, spouse caregivers	(a) The care recipients were diagnosed with dementia, (b) Caregivers identified themselves as the primary caregiver or shared an equal caregiving burden with other major caregivers, and (c) caregivers were 18 years old or older.	The Marwit and Meuser Caregiver Grief Inventory – Short Form	The Chinese Version of Multidimensional Scale of Perceived Social Support (MSPSS): Family subscale.	Family support significantly decreased PDG
Cheng et al. 2019	HK, China	Quantitative design	N = 173; 79% female; mean age: 63.8 years ± 10.5	Purposeful sampling, children, spouse caregivers	Primary family caregiver provided more than 14 hours of care per week for AD patients over 60.	Caregiver Grief Questionnaire	Medical Outcomes Study Social Support Survey	Social network size was not found to be associated with grief
Kobiske et al. 2019	USA	Quantitative design	N = 104; 65.26% females; mean age: 58.27 (11.21)	Convenience sample children, spouse caregivers	Caring for a partner/spouse) with a diagnosis of young-onset dementias (YODs)	Marwit‐Meuser Caregiver Grief Inventory Short Form	Resourcefulness Scale	Personal resourcefulness did not moderate the relationship between stress and PDG while social resourcefulness moderated this relationship.
Manevich et al. 2023	Israel	Quantitative design (RCT)	N = 94; 41.49% females; mean age: 71.43 (10.50)	Convenience sample, spouse caregivers	Caring for partner/spouse) with a diagnosis of young-onset dementias	Two-Track Dementia Grief Questionnaire (TTDG-Q).	Medical Outcomes Study Social Support Survey (MOSSS). Sense of Coherence Inventory (SOC-S). Experiences in Close Relationships Scale (ECR-S).	Lower levels of sense of coherence and tangible social support were related to higher levels of grief.
Meichsner et al. 2016	Germany	Quantitative design	N = 229; 79% female; mean age: 63.8 years ± 10.5	Purposeful sampling, children, spouse caregivers	Home-caregivers of a family member with dementia	Caregiver Grief Scale (CGS)	Social Relationships of WHO Quality of Life-BREF–German Version (WHOQoL-BREF).	Greater satisfaction with social relationships was related to lower grief
Moore et al. 2017	UK	Mixed-method design	N = 35; 68.57% females;	Purposeful sampling, children, spouse caregivers, relatives	People caring for someone with a diagnosis of dementia	Inventory of Complicated Grief (ICG) Short-Form Pre-Loss version	Brief Coping Orientation to Problems Experienced (Brief‐COPE) questionnaire	There was less use of the coping strategies: instrumental support, substance use, denial and planning. The most commonly used strategy was humor, followed by seeking emotional support and acceptance.
			N = 12; 66.67% females; mean age: 43 − 91 years old	Purposeful sampling, 8 daughters, 2 sons and 2 husbands	People caring for someone with a diagnosis of dementia			Results indicated the importance of relationships with health and social care services, understanding of the progression of dementia, and emotional responses to advanced dementia; the carer’s perception of their ability to control and influence end of life care; social networks from friends, family and faith community
Moore et al. 2020	UK	Quantitative design	N = 150; 77.3% females; mean age: 63.0 (12.1)	Purposeful sampling, spouse, adult children, others	Eligible caregivers provided practical, social, emotional or supervisory support; aged 18 and over.	Marwit‐Meuser Caregiver Grief Inventory Short Form	Duke University Religion Index; Health Literacy Questionnaire; Relationship Closeness Scale; Social Support subscale of the HLQ; Dementia Knowledge Assessment Scale	Reduced social support was strongly associated with higher grief intensity along with the confounders of female gender, spouse or adult child relationship type and reduced relationship closeness. In exploratory analyses of MMCGI-SF sub-scales, higher dementia knowledge was associated with lower “heartfelt sadness.”
Moore et al. 2023	Australia	Mixed-method	N = 150; 77% females; mean age: 63(12.1)	Purposeful sampling, children, spouse caregivers	People caring for someone with a diagnosis of dementia	Marwit‐Meuser Caregiver Grief Inventory Short Form	Brief Coping Orientation to Problems Experienced (Brief‐COPE) questionnaire	Emotional-oriented coping was negatively associated with PDG and dysfunctional coping was also negatively associated with higher PDG, with a small negative association with problem‐focused strategies
				Purposeful sampling, children, spouse caregivers				Qualitative themes matched the contents of Brief-COPE. Unhelpful strategies of denial and avoidance align with dysfunctional coping strategies. Psychological strategies (including acceptance and humor) and seeking support were consistent with emotion‐focused strategies, but we did not identify a theme relating to problem‐focused strategies.
Popok et al. 2022	USA	Qualitative design	N = 17; 52.9% females; mean age: 61.47 (5.23)	Purposeful sampling, partner caregivers	People caring for partners with a diagnosis of dementia			Caregivers reported the understanding of illness could reduce frustration and connect with partner. They tried to focus on the present with gratitude and patience. In addition, they try to connect with social support and accept help. Most importantly, they practice self-care and patience to manage stressors.
Rigby et al. 2021	USA	Quantitative design	N = 515; 90.1% females; mean age: 61.47 (5.23)	Purposeful sampling, DLB, PDD, AD caregivers	People caring for partners with a diagnosis of DLB, PDD, AD	Marwit‐Meuser Caregiver Grief Inventory Short Form	Dementia Care Confidence scale; 12investigator-generated caregiver mastery and self-efficacy measurement	Caregivers’ self-efficacy, mastery and confidence was positively related to grief.
Romero et al. 2014	USA	Quantitative design	N = 66; Mean age: 64.3 (7.6)	Convenience sampling spouse and children caregivers	(a) being a spouse or adult child of a person with a diagnosis of Alzheimer’s disease and (b) being a primary or secondary caregiver who provides a broad range of assistance consisting of toileting, dressing, eating and assistance with other activities of daily living.	Marwit‐Meuser Caregiver Grief Inventory Short Form	Brief Cope Inventory; the Inventory of Social Support	Dysfunctional coping increased PDG while social support decreased PDG.
Wangliu and Chen 2024	China	Quantitative design	N = 320; 52.5% females; mean age: 54.706 (4.029)	Convenience sampling; children caregivers	People caring for partners with a diagnosis of dementia	Marwit‐Meuser Caregiver Grief Inventory Short Form	Ways of Coping Checklist-Revised (WCCLR)	Caregiving burden prevented the use of active coping, and this decrease in coping increased the perception of pre-death grief.

### Quality appraisal

We utilized the 2018 edition of the Mixed Methods Appraisal Tool (MMAT) to conduct a rigorous evaluation of the quality of quantitative, qualitative, and mixed methods research papers (Hong 2018). Through the application of MMAT, we scrutinized various aspects such as the appropriateness of study designs, methods of data collection, results interpretation, and consistency in qualitative research; representation of samples, control of confounding variables, measurement techniques, and appropriateness of interventions in quantitative research; as well as the coherence of mixed-method designs, integration of qualitative and quantitative elements, and congruity of findings in mixed-method studies. The evaluation process involved two independent assessments by the authors, with discrepancies being resolved through deliberation and, if necessary, consultation with a third reviewer, mirroring the screening methodology employed for the selection of studies. Of the 12 included studies, they met all the methodological quality criteria of the MMAT in Table 2.

**Table 2.** Quality evaluation of selected articles

Qualitative articles	Are there clear research questions?	Do the collected data allow to address the research questions?	Is the qualitative approach appropriate to answer the research question?	Are the findings adequately derived from the data?	Is the interpretation of results sufficiently substantiated by data?	Is there coherence between qualitative data sources, collection, analysis and interpretation?	Is there clear mention of ethical approval process in the article?
Popok et al. 2022	✓	✓	✓	✓	✓	✓	✓
Quantitative articles	Are there clear research questions?	Do the collected data allow to address the research questions?	Is the sampling strategy relevant to address the research question?	Is the sampling representative of the target population?	Are the measurements appropriate?	Is the risk of non-response bias low?	Is the statistical analysis appropriate to answer the research question?
Chan et al. 2017	✓	✓	✗	✓	✓	✓	✓
Cheng et al. 2019	✓	✓	✓	✓	✓	✓	✓
Kobiske et al. 2019,	✓	✓	✓	✓	✓	✓	✓
Manevich et al. 2023	✓	✓	✓	✓	✓	✓	✗
Meichsner et al. 2016	✓	✓	✗	✓	✓	✓	✓
Moore et al. 2020	✓	✓	✓	✓	✓	✓	✓
Rigby et al. 2021	✓	✓	✓	✗	✓	✓	✓
Romero et al. 2014	✓	✓	✓	✓	✓	✗	✓
Wangliu and Chen 2024	✓	✓	✓	✓	✓	✓	✓
Mixed methods	Are there clear research questions?	Do the collected data allow to address the research questions?	Is there an adequate rationale for using a mixed-methods design to address the research question?	Are the different components of the study effectively integrated to answer the research question?	Are the outputs of the integration of qualitative and quantitative components adequately interpreted?	Are divergences and inconsistencies between quantitative and qualitative results adequately addressed?	Do the different components of the study adhere to the quality criteria of each tradition of the methods involved?
Moore et al. 2017	✓	✓	✓	✓	✓	✓	✗
Moore et al. 2023	✓	✓	**✓**	✓	✓	✓	✓

## Results

### Coping strategies and PDG

Five papers examined the coping strategies employed by family caregivers to mitigate PDG during their caregiving processes. The ongoing debate regarding the categorization of coping strategies complicates the comparison, contrast, and synthesis of findings from the studies reviewed. For instance, some research (Moore et al. 2023; Romero et al. 2014) has distinguished between emotion-focused, problem-focused, and dysfunctional coping strategies. Their findings indicate that emotion-focused and problem-focused styles are associated with lower levels of grief, whereas dysfunctional coping styles are linked to elevated grief levels. Moore et al. (2023) also conducted interviews which revealed that negative strategies such as denial and avoidance correlate with dysfunctional coping approaches. Two prominent themes identified in our analysis – psychological strategies including acceptance and humor, and the pursuit of support – are closely aligned with emotion-focused coping strategies. Specifically, interviews indicated that caregivers employed a variety of cognitive coping strategies to reframe their circumstances, enhancing manageability. These strategies encompassed acceptance, perseverance, maintaining a positive and pragmatic outlook, and using humor. Notably, these approaches corresponded with the emotion-focused coping strategies assessed by the Brief-COPE scale, which were associated with lower grief levels. Furthermore, caregivers reported actively seeking both formal and informal social support systems as a means of coping with their grief, underscoring their recognition of the importance of external assistance in processing emotions and navigating challenges.

In another study, Moore et al. (2017) incorporated the Brief COPE scale to assess coping strategies utilized by participants. The findings indicated a reduced utilization of coping strategies such as instrumental support, substance use, denial, and planning. Conversely, some emotional coping strategies such as humor emerged as the most frequently employed coping strategy, followed by seeking emotional support and acceptance. Subsequently, the researchers conducted interviews to delve deeper into the participants’ experiences. During these interviews, it became evident that accessing health and social care services played a crucial role in effective coping. Additionally, having a comprehensive understanding of the progression of dementia was highlighted as an important factor in navigating the challenges associated with the condition.

A recent article by Wangliu and Chen (2024) utilized the Ways of Coping Checklist-Revised and found that caregiving burden can diminish the utilization of active coping strategies, which include problem-focused coping and seeking social support. This decrease in active coping subsequently contributes to increased levels of PDG. Additionally, a study involving interviews conducted by Popok and colleagues (2022) revealed that caregivers reported that understanding the illness helped to mitigate frustration and strengthen their connection with their partners. They emphasized the importance of focusing on the present while cultivating gratitude and patience. Furthermore, caregivers sought connections with social support networks and accepted assistance. Crucially, they prioritized self-care and patience as means to effectively manage stressors. Rigby et al. (2021) discovered that caregivers place importance on various adaptive coping strategies. These strategies include gaining knowledge about the diagnosis, utilizing social resources, maintaining a positive outlook, seeking social support, and practicing self-care. Notably, Kobiske and colleagues (2019) found that personal resourcefulness, which pertains to one’s ability to maintain daily independence and self-help, did not act as a moderator in the relationship between predeath grief and perceived stress. On the other hand, social resourcefulness, which involves seeking support from others, did demonstrate a positive moderating effect on this relationship.

In general, results of studies revealed that among different coping strategies, active or problem-focused strategies such as seeking social support from professional services or informal social networks and finding information about illness or treatments could alleviate caregivers’ grief. Some emotional coping strategies such as avoidance or self-blaming and dysfunctional coping might not be helpful. In addition, maintain independence, self-care and humor are useful strategies for caregivers to maintain their mental status.

### Coping resources and PDG

As for the personal resources, self-efficacy and sense of coherence are the essential resource to decrease PDG. Rigby and colleagues (2021) found self-efficacy could alleviate grief. When caregivers have belief and confidence to deal with caregiving tasks, their sense of loss will decrease. Manevich and colleagues (2023) found that sense of coherence could also be helpful. If caregivers are more resilient to stressors in daily life, they will stay well and their health will be improved. Interviews by Moore and colleagues (2023) found caregivers decision-making is important because whether they could be informed about dementia and make a decision for care recipients to admission to care home is relevant to their perception of grief.

Social support is a critical resource for caregivers of individuals with dementia. While the size of one’s social network has not been found to correlate with levels of grief (Cheng et al., 2019), studies have identified negative associations between grief and social support, encompassing both informal and formal forms of assistance (Chan et al. 2017; Romero et al. 2014). For instance, informal social support from friends, family, and the community could decrease caregivers’ perception of grief (Moore et al. 2023, 2020, 2017). Specifically, formal support which includes carer support groups, information delivering services, physicians, or counselling services could be related to their perception of grief (Moore et al. 2023). Furthermore, a correlation was identified between higher satisfaction with social relationships and reduced levels of grief (Meichsner et al. 2016). If caregivers are more satisfied with their relationship with care recipients, they are more likely to obtain gains rather than losses in the caregiving role. Instead, according to Moore et al. (2020, 2023), the inability to seek help due to troubled past relationships with other family members worsened emotional distress and frustration. Carers were disappointed and angered by family and friends who withdrew from them or disagreed with their caregiving methods in relation to the person with dementia. These experiences further highlighted the necessity of establishing a dependable support system.

Whether caregivers have spiritual religious belief and community faith (Moore et al. 2023) is essential to alleviate their grief. For instance, if they have spiritual faith, they will believe that they would conquer difficulties. At the same time, gathering with others could also bring them a sense of support.

## Discussion

This review integrates data from a vast and diverse array of international literature aimed at addressing two primary research inquiries. The first question pertains to the relationship between coping strategies and PDG. Generally, the studies revealed that the utilization of coping strategies is effective in mitigating PDG and alleviating the impact of caregiving burden on PDG. Specifically, employing problem-focused coping strategies to adapt to the caregiving role and responsibilities was linked to lower levels of PDG. Furthermore, maintaining independence, self-care, and humor were identified as beneficial strategies for caregivers in preserving their mental well-being. Most emotion-focused coping strategies exhibited a negative correlation with PDG, except for avoidance or self-blaming strategies, which may exacerbate caregivers’ PDG.

Nevertheless, certain anecdotal evidence from qualitative data highlighted the utility of some emotion-focused techniques, underscoring the dynamic and evolving nature of coping mechanisms and the importance of considering individual contexts. Previous research has highlighted that coping strategies cannot be strictly classified as either positive or negative. Certain stressors may be more effectively managed using emotion-focused techniques, especially when they are resistant to modification through problem-focused approaches (Lazarus and Folkman 1984). Regrettably, only limited information is available in the reviewed literature regarding the alignment between stressors and specific coping strategies chosen by caregivers, necessitating further investigation.

Alongside problem- and emotion-oriented strategies, a number of cognitive coping mechanisms have also been recognized. These approaches entail a deliberate endeavor to modify the perceptions, evaluations, or cognitive processes associated with caregiving, with the aim of enhancing overall well-being (Hawken et al. 2018). In contrast to problem-focused or emotion-focused coping, cognitive coping are non-behavioral and are characterized as mental constructs employed to address stressful or challenging circumstances. These strategies are fundamentally concerned with the individual’s mental assessment of their capacity to manage a stressor (Hawken et al. 2018; Lazarus and Folkman 1984). While cognitive elements may be viewed as separate from problem- and emotion-oriented coping, it is vital to acknowledge the interconnectedness of these three categories of coping strategies. The interactions among various coping styles highlighted in this review are consistent with earlier studies, which suggest that an integrated approach combining problem-focused, emotion-focused, and cognitive strategies generally proves to be the most effective method for managing stressors (Pakenham 2001). Nonetheless, the research included in this review does not provide insights into the ideal equilibrium among different coping methods or the range of coping strategies utilized by caregivers.

The second question explored how coping resources were associated with PDG. Coping resources, such as self-efficacy, sense of coherence, and support from friends and family, are likely to have a reducing effect on PDG. The quality of relationships with friends and family members also plays a crucial role. Caregivers who report dissatisfaction with their relationships with friends and family tend to experience higher levels of PDG during dementia caregiving. Furthermore, spiritual and religious beliefs as well as community faith have been identified as essential factors in alleviating grief among caregivers (Moore et al. 2023).

Caregivers with initially higher levels of SOC tend to exhibit even lower levels of PDG compared to those with lower SOC levels. According to Antonovsky (1987), individuals with a strong SOC possess greater coping capabilities and more available resources, enabling them to perceive situations as more comprehensible and meaningful, thus facilitating better resource management.

In terms of family support, both instrumental and emotional support from family members are crucial for Chinese elderly populations (Yeung and Fung 2007). Studies have shown that receiving substantial support from a spouse or siblings is linked to positive self-assessment, reduced feelings of loneliness, and enhanced overall well-being (Thomas 2010). Nevertheless, an overly abundant provision of support from others may hinder the effective coping efforts of caregivers (Huang et al. 2009). For instance, caregivers who receive extensive family support may expend significant energy on caregiving tasks and coping strategies, potentially neglecting their self-care needs, leading to detrimental effects on their own PDG.

According to the stress -process model, coping resources and coping strategies have positive effects on well-being, irrespective of the level of stress (Cohen & Wills 1985). An understanding of the determinants of individual coping strategies lies with knowledge about their appraised stress, their personal and social resources, and their coping responses (Lazarus and Folkman 1984). With rich access of resources and the abilities of active coping strategies could contribute to wellbeing of caregivers (Pinquart et al., 2004).

Although this research presents a current analysis of the coping factors that play a pivotal role in the grief experience, some limitations exist. While the studies reviewed encompassed various nations, there was a notable lack of representation from lower middle-income countries. Nevertheless, a growing body of research from diverse cultural backgrounds has emerged, highlighting variations in coping mechanisms and grief experiences. It is important to note that generalizing findings across the included studies may pose challenges due to the diverse caregiving systems, cultural contexts, study methodologies, target populations, and assessments of factors influencing PDG in Western and Asian settings. A factor that influences one setting may not have the same impact in another setting. For instance, in China where family caregiving is highly demanded, social support resources may be more prominent, whereas in Western countries, the availability of community services resources could be more crucial. There is a dearth of longitudinal studies exploring the progression of grief over time or during bereavement, which could provide a deeper insight into the coping process during mourning. Furthermore, it is worth noting that this review only considered English-language studies based on our inclusion criteria. This exclusion may have led to the oversight of significant studies published in languages other than English, particularly in Asian regions. Based on these limitations, future studies might focus on exploring coping and PDG in lower middle-income countries. Longitudinal studies should be considered to the process of grief. Moreover, comparative studies across nations might be considered to explore relevant associations in diverse cultural context.

This study offers important implications for practice. Understanding the impact of coping mechanisms on the grief process can be vital in identifying individuals who are vulnerable to elevated levels of PDG. This comprehension entails examining how various coping strategies, whether adaptive or maladaptive, influence an individual’s emotional response to PDG. For instance, individuals who engage in positive coping strategies – such as seeking social support or utilizing mindfulness techniques – may navigate their grief more effectively (Paun et al. 2019). They may openly discuss their feelings with friends and family, participate in support groups, or engage in activities that commemorate their loved ones. Consequently, they might demonstrate a sense of calm and acceptance while coping with the uncertainties associated with a loved one’s illness, resulting in a more manageable grief experience. In contrast, individuals who resort to negative coping mechanisms, such as avoidance, substance misuse, or excessive denial, may encounter intensified levels of PDG. For example, individuals who avoid discussing their terminally ill loved ones or suppress their emotions may experience profound sadness (Moore et al. 2023). This accumulation of unprocessed emotions could lead to an overwhelming reaction upon the actual loss, potentially exacerbating their grief. By recognizing these patterns, mental health professionals and caregivers can develop interventions that promote healthier coping strategies. As social support resources impose an effect on PDG, it is essential to establish family support programs that include counseling and skill-building workshops to improve family dynamics and communication during the caregiving process and create support networks within families and communities to share experiences and resources, which can mitigate grief.

## Conclusion

The results suggest that coping strategies and available resources significantly influence the grieving process of family caregivers for individuals with dementia. Recognizing these factors, which can elevate the probability of experiencing PDG, is essential for identifying those who may require assistance. Future studies should investigate the interactions among these elements. Furthermore, there is a scarcity of evidence concerning service utilization in relation to PDG, and subsequent research should also examine which aspects of support or service delivery are most critical for caregivers dealing with PDG.
