# A systematic review of coping and pre-death grief among dementia family caregivers – CORRIGENDUM

**DOI:** 10.1017/S1478951525100357

**Published:** 2025-08-07

**Authors:** Yi-Qi Wangliu, Run-Ping Che

**Affiliations:** 1 School of Ethnology and Sociology, Yunnan University, Kunming, mainland, China; 2 Department of Social Work, The Chinese University of Hong Kong, Shatin, NT, Hong Kong SAR, China

**Keywords:** Dementia, Caregivers, Grieving, Palliative care, Coping, Corrigendum

The Publisher apologises for an error in the author affiliation information, but this has now been corrected in the published article.

Yi-Qi Wangliu is affiliated only with: School of Ethnology and Sociology, Yunnan University, Kunming, mainland, China.

Run-Ping Che is affiliated with: Department of Social Work, The Chinese University of Hong Kong, Shatin, NT, Hong Kong SAR, China
